# Fractures among patients with dizziness – a ten-year follow-up

**DOI:** 10.1186/s12877-018-0734-2

**Published:** 2018-02-02

**Authors:** Eva Ekvall Hansson, Anders Beckman

**Affiliations:** 1 Department of Health Sciences, Physiotherapy, Lund University, HSC, Baravägen 3, SE221 00 Lund, Sweden; 2 Department of Clinical Sciences in Malmö/General Practice, Lund University, Malmö, Sweden

**Keywords:** Dizziness, Falls, Fall prevention, Fracture

## Abstract

**Background:**

The number of elderly people persons suffering from dizziness is substantial, and dizziness is a risk factor for falls and fractures. Fall-related fractures represent a major public health issue. Longitudinal studies can help find ways of predicting fall-related fractures among frail elderly persons with multisensory dizziness. The aim of the present study was therefore to investigate whether different measures of balance, being male/female or admission to hospital, could predict fracture at a ten-year follow-up in patients suffering from multisensory dizziness.

**Methods:**

Patients who had participated in two earlier (ten years previous) dizziness studies were sought in the local health authority’s patient administrative system. Information was extracted regarding patient hospitalization, for fractures or for any other reason, during the ten-year period. Logistic regression was used to analyse the relations between clinical balance measures, vestibular rehabilitation, admission to hospital, sex, and fracture.

**Results:**

There was no difference between the group of patients with fracture and the group of patients without fracture, regarding balance measures at baseline or admission to hospital for reasons other than fracture. There was no difference between men and women in any of the measures.

**Conclusions:**

This study did not identify any predictors of fracture. Thus, among frail elderly, attention to fall risk should be equally high regardless of patient history.

## Background

Falls and fall-related accidents represent a major Swedish public health issue, accounting for 70% of all in-hospital treatments due to accidents [1]. In Sweden, the annual cost of falls among older people is approximately 14 billion Swedish kroner (1.5 billion USD), and considerable gains could be made by a decline in the number of falls, both for the persons and the society [1].

Most often older people fall while doing everyday activities, such as taking a walk, rising from or sitting down at a chair, or simply just shifting body weight while doing other activities [2, 3].

Impaired postural stability is common among elderly people with low-energy distal radius fractures [4], and asymmetric vestibular function is overrepresented in elderly persons with hip fractures [5] or wrist fractures [6, 7]. Vestibular asymmetry can also predict falls among elderly people with dizziness [8] and can be affected positively by vestibular rehabilitation [6].

The number of elderly people suffering from dizziness is substantial [9]. Dizziness is a risk factor for falls [10], and has been shown to increase the risk of non-osteoporotic fractures [11]. The cause of dizziness among elderly persons is often benign and seldom life-threatening [12] and a multifactorial cause can often be assumed [13]. Dizziness with a multifactorial cause is attributed to increasing age and deterioration of multiple sensory receptor systems, and is defined as multisensory dizziness [13, 14]. A typical feature of this condition is problems with walking [15].

We have been studying elderly with multisensory dizziness in a series of studies. In two different studies we showed that vestibular rehabilitation improved balance [16, 17]. A five-year follow-up study including persons from these studies showed that persons with multisensory dizziness who were admitted to hospital for any reason, were at high risk of suffering from any kind of fracture [18]. However, none of the balance measures from baseline or after vestibular rehabilitation at the five-year follow-up could predict falls and consequent fractures. A longer follow-up might help find ways of predicting fall-related fractures among this group of frail elderly.

The aim of the present study was therefore to investigate whether different measures of balance, being male/female or admission to hospital, could predict fracture at a ten-year follow-up in patients suffering from multisensory dizziness.

## Methods

### Participants

Letters were sent to patients from the two previous studies [16], with an invitation to participate in this longitudinal study. Searches in the local health authority’s patient administrative system (PAS) were performed, ten years after baseline measures in the two previous studies (*n* = 108) [16], excluding those patients who declined participation (*n* = 6), giving a sample size of 102. Patients, who had deceased since baseline measures, were included without informed consent from next of kin, which is according to Swedish law. Information was extracted about whether the patient had been hospitalized, for a fracture or any other reason, during the ten-year period.

In study 1 [16] the baseline measures for balance were:

- The Romberg test, standing with feet together for 60 s, first with eyes open and then with eyes closed [19]. Time in seconds was noted.
- Standing one leg eyes open (SOLEO) and standing one leg eyes closed (SOLEC) for 30 s [20].
- Walking heel to toe for 5 m on a 5-cm-wide line marked on the floor [21]. The number of steps outside the line was noted.
- Walking in a figure of eight, with the number of steps outside the figure being noted [22].

In study 2 the baseline measures for balance were:

- Tandem standing with eyes open and with eyes closed for 30 s [23].
- Standing one leg eyes open (SOLEO) and standing one leg eyes closed (SOLEC) for 30 s [20].
- Walking heel to toe for 5 m on a 5-cm-wide line marked on the floor [21]. The number of steps outside the line was noted.
- Walking in a figure of eight, with the number of steps outside the figure being noted [22].

### Statistics

To analyse variables of potential significance for fracture, several methods were used. Because of the characteristics of the variables (non-normal distribution, restricted values with cut-off (Romberg/Tandem-Romberg)), we employed the non-parametric Mann-Whitney test.

Binary outcome of fracture was analysed with multivariate logistic regression. The outcome variable was fracture of any kind (yes vs. no) and the independent variables were Romberg/Tandem Romberg, SOLE (with eyes both open and closed), walking heel-to-toe, walking in a figure of eight and vestibular rehabilitation (yes vs. no). To analyse differences between groups, a chi-square test was used for fracture vs. sex, death, and admission to hospital for reasons other than fracture.

### Ethics

The study was approved by the regional ethical review board in Lund (2014/361).

## Results

Six patients declined the searches in the databases, thus the total study sample consisted of 102 patients (63 still alive and 39 who had died during the study period). There were 70 women and 32 men included. Forty-three patients underwent vestibular rehabilitation during the initial study period 10 years earlier. A total of 42 patients sustained one or more fractures that resulted in a visit or admission to hospital during the follow-up period. There was no difference between the group of patients with fracture and the group of patients without fracture, regarding balance measures at baseline, vestibular rehabilitation or admission to hospital for reasons other than fracture (*p* = 0.26–0.89) (Table 1). There was no difference between men and women in fractures (*p* ≥ 0.26), data not shown. In the logistic regression, no independent variable was associated with fracture (Table 2).

**Table 1:** Baseline data of the population and follow-up data divided into the group with no fractures and the group with fractures

Measures	All at baseline *n* = 102	No fracture at follow-up *n* = 60	Fracture at follow-up *n* = 42	*p*-value
Age	78.7	77.9	80	0.318
Women/men (n)	70/32	38/22	32/10	0.168
Deceased (n)	73 (72%)	39 (65%)	34 (81%)	0.079
Romberg eyes open *n* = 50 (s)	56.0	56.9	54.8	0.562
Romberg eyes closed *n* = 50 (s)	48.2	45.9	51.1	0.370
Tandem Romberg eyes open *n* = 53 (s)	16.0	17.2	14.0	0.377
Tandem Romberg eyes closed *n* = 53 (s)	3.5	4.2	2.5	0.397
SOLEO (s)	8.2	7.8	8.7	0.628
SOLEC (s)	1.4	1.4	1.5	0.753
Figure of eight (number of mistakes)	13.0	12.8	13.2	0.896
Walking heel to toe (number of mistakes)	6.8	7.3	6.0	0.260
Admitted to hospital for other reason than a fracture (n)	90	52	38	0.556

**Table 2:** Logistic regression of associations with fracture. Fracture as dependent outcome

Independent variable	OR	95% CI
Romberg OE*	0.93	0.86–1.01
Romberg CE*	1.05	0.99–1.11
Tandem Romberg OE*	0.99	0.94–1.04
Tandem Romberg CE*	0.97	0.88–1.08
SOLE OE*	1.01	0.95–1.07
SOLE CE*	0.99	0.76–1.29
Walking h-t-t	0.94	0.85–1.04
Walking 8	1.01	0.97–1.06
Westibular rehab	0.73	0.33–1.64

*OE open eyes, *CE* closed eyes

## Discussion

This study showed that in this study sample consisting of patients with multisensory dizziness, none of the balance measures used ad baseline could predict fracture in a ten-year follow-up. Admission to hospital for any reason has shown to predict fracture in a five-year follow-up of the same study sample but this was not the case in this ten-year follow-up.

The register information (PAS) used in this study is used for resource allocation and has been checked for registration errors by professional experts, and must therefore be judged as highly reliable. Since we have a long follow-up period, these results can probably be generalized.

We have followed the same sample of patients for both five and 10 years. At the 5 year follow-up, we found that admission to hospital, for any reason other than fracture, was associated with having a fracture [18] but this association was lost at the ten-year follow up. The study group consist of elderly with balance disorders, which is a risk factor for falls. Hence, the risk of a fall-related fracture 10 years after baseline measures is probably very high, which might explain the loss of association. Also, we have no information of the impact of other diseases, besides multisensory dizziness, on the outcome. Thus, in a short perspective, attention to fall risk among elderly persons with dizziness admitted to hospital for any reason should be high. However, the prevention of falls is complex and in a long perspective, special attention to fall risk is probably beneficial to all frail elderly not only this specific group of patients.

Standing one leg and gait speed has shown to be able to predict fracture in a ten-year follow-up study with a larger sample size than in our study [23]. Combining balance measures with a cognitive task or with other fracture risk assessments, such as FRAX® might also increase the possibility to predict fracture [24, 25]. So far, previous fall seems to be the strongest predictor of a future fracture [2]. Thus, it seems important to identify factors that can predict the first fall, in order to predict future fracture.

## Conclusions

This study did not identify any balance measures that could predict fracture. Thus, among frail elderly, attention to fall risk should be equally high regardless of patient history.

## Abbreviations

PAS: Patient administrative system
SOLEC: Standing one leg eyes closed
SOLEO: Standing one leg eyes open

## References

[1] The National Board of Health and Welfare. Injuries among elderly in Sweden 2014 (in Swedish) (online). Available at: www.socialstyrelsen.se: Socialstyrelsen; Accessed 23 Jan 2017.

[2] Gillespie LD, Robertson MC, Gillespie WJ, Sherrington C, Gates S, Clemson LM (2012). Interventions for preventing falls in older people living in the community. Cochrane Database Syst Rev.

[3] Robinovitch SN, Feldman F, Yang Y, Schonnop R, Leung PM, Sarraf T (2013). Video capture of the circumstances of falls in elderly people residing in long-term care: an observational study. Lancet.

[4] Louer CR, Boone SL, Guthrie AK, Motley JR, Calfee RP, Wall LB (2016). Postural stability in older adults with a distal radial fracture. J Bone Joint Surg Am.

[5] Kristinsdottir EK, Jarnlo G-B, Magnusson M (2000). Asymmetric vestibular function in the elderly might be a significant contribution to hip fractures. Scand J Rehab Med.

[6] Ekvall Hansson E, Dahlberg LE, Magnusson M (2015). Vestibular rehabilitation affects vestibular asymmetry among patients with fall-related wrist fractures - a randomized controlled trial. Gerontology.

[7] Kristinsdottir EK, Nordell E, Jarnlo GB, Tjäder A, Thorngren KG, Magnusson M (2001). Observation of vestibular asymmetry in a majority of patients over 50 years with fall-related wrist fractures. Acta Otolaryngol.

[8] Ekvall Hansson E, Magnusson M (2013). Vestibular asymmetry predicts falls among elderly patients with multi- sensory dizziness. BMC Geriatr.

[9] de Moraes SA, Soares WJ, Ferriolli E, Perracini MR (2013). Prevalence and correlates of dizziness in community-dwelling older people: a cross sectional population based study. BMC Geriatr.

[10] Tuunainen E, Rasku J, Jantti P, Pyykko I (2014). Risk factors of falls in community dwelling active elderly. Auris Nasus Larynx.

[11] Kruschinski C, Sheehy O, Hummers-Pradier E, Lelorier J. Fracture risk of patients suffering from dizziness: a retrospective cohort study. Eur J Gen Pract. 2010;16(4):229–35. 10.3109/13814788.2010.517630. Epub 2010 Sep 19. 10.3109/13814788.2010.517630 20849315

[12] Kroenke K, Lucas CA, Rosenberg ML, Scherokman B, Herbers JE, Wehrle PA (1992). Causes of persistent dizziness. Ann Intern Med.

[13] Kao AC, Nanda A, Williams CS, Tinetti ME (2001). Validation of dizziness as a possible geriatric syndrome. J Am Geriatr Soc.

[14] Wetmore SJ, Eibling DE, Goebel JA, Gottshall KR, Hoffer ME, Magnusson M (2011). Challenges and opportunities in managing the dizzy older adult. Otolaryngol Head Neck Surg.

[15] Hansson EE, Månsson N-O, Håkansson A (2004). Assessment and management of vertigo and dizziness among older persons. Rev Clin Gerontol.

[16] Hansson EE, Månsson NO, Håkansson A (2004). Effects of specific rehabilitation for dizziness among patients in primary health care. A randomized controlled trial. Clin Rehabil.

[17] Hansson EE, Månsson N-O, Håkansson A, Ringsberg KA (2008). Falls among dizzy patients in primary health care - an intervention study with control group. Int J Rehabil Res.

[18] Beckman A, Hansson EE (2011). Fractures in people with dizziness: 5-year follow-up. J Am Geriatr Soc.

[19] Ringsberg KAM, Gärdsell P, Johnell O, Jónsson B, Obrant KJ, Sernbo I (1998). Balance and gait performance in an urban and a rural population. JAGS.

[20] Jarnlo G-B, Thorngren K-G (1991). Standing balance in hip fracture patients. Acta Orthop Scand.

[21] Ledin T, Kronhed AC, Möller C, Möller M, Ödkvist LM, Olsson B (1990). Effects of balance training in elderly evaluated by clinical tests and dynamic posturography. J Vestib Res.

[22] Johansson G, Jarnlo G-B (1991). Balance training in 70-year-old women. Physiother Theory Pract.

[23] Wihlborg A, Englund M, Åkesson K, Gerdhem P (2015). Fracture predictive ability of physical performance tests and history of falls in elderly women: a 10-year prospective study. Osteoporos Int.

[24] Najafi DA, Dahlberg LE, Hansson EE (2016). A combination of clinical balance measures and FRAX (R) to improve identification of high-risk fallers. BMC Geriatr.

[25] Nordin E, Moe-Nilssen R, Ramnemark A, Lundin-Olsson L (2010). Changes in step-width during dual-task walking predicts falls. Gait Posture.

